# A new fossil silky lacewing genus (Neuroptera, Psychopsidae) from the Early Cretaceous Yixian Formation of China

**DOI:** 10.3897/zookeys.130.1576

**Published:** 2011-09-24

**Authors:** Yuanyuan Peng, Vladimir N. Makarkin, Xiaodong Wang, Dong Ren

**Affiliations:** 1 College of Life Sciences, Capital Normal University, 105 Xisanhuanbeilu, Haidian District, Beijing, 100048, China; 2 Institute of Biology and Soil Sciences, Far Eastern Branch of the Russian Academy of Sciences, 100 let Vladivostoku Street 159, Vladivostok, 690022, Russia; 3 Administration Office, Liaoning Chaoyang Bird Fossil National Geopark, 100 Longniao Street, Longcheng District, Chaoyang City, Liaoning Province, 122000, China

**Keywords:** Psychopsidae, Osmylopsychopidae, fossil, Mesozoic, Huangbanjigou, China

## Abstract

A new genus and species, *Undulopsychopsis alexi* **gen. et sp. n.,** is described from the Early CretaceousYixian Formation of western Liaoning Province, China. This genus is probably most closely related to the Asian Cretaceous genus *Kagapsychops* Fujiyama, 1978. The family affinity of *Undulopsychopsis* **gen. n.** is discussed. The genus is preliminarily assigned to Psychopsidae, although it shares some character states with Osmylopsychopidae (e.g., crossveins are very scarce; Rs1 and 1A are multi-branched).

## Introduction

The extant Psychopsidae is a small family (five genera and 27 described species), currently restricted to disjunct regions in southern Africa, southeastern Asia and Australia (New 1988; Oswald 1993a; Wang and Bao 2006). Adult psychopsids are recognized by their broad wing shape, dense venation, the presence of *vena triplica*, spectacularly patterned and hairy wings, which gives psychopsids the common name of silky lacewings (New 1989; Oswald 1993a, 1995).

Fossil psychopsids were much more widely distributed than the extant taxa. Since the early 20th century, fossil psychopsids have increasingly been found from all over the world, with species ranging in age from the Triassic to the Tertiary. The earliest fossil record of the Psychopsidae is *Triassopsychops* Tillyard, 1922 from the Late Triassic of Australia (Tillyard 1922), which possesses a true *vena triplica*, characteristic of this family. While many fossil psychopsids were recorded from the Mesozoic, few representatives have been described from the Tertiary. Hitherto, 35 fossil species (24 genera) have been referred to Psychopsidae (Table 1). The psychopsid affinity of many Jurassic and Cretaceous genera is debatable. For example, Jepson et al. (2009) believe that some genera (e.g., *Grammapsychops* Martynova, 1954, *Embaneura* G. Zalessky, 1953, *Pulchroptilonia* Martins-Neto, 1997, and *Kagapsychops* Fujiyama, 1978) may belong to another psychopsoid family, Osmylopsychopidae. The position of these and some other fossil genera from the Mesozoic is questionable, due to certain differences in details of the wing venation between fossil and extant psychopsids (e.g., the pattern of Rs branches, the configuration of M and Cu in the forewing, the structure of *vena triplica*), although their general venational pattern is similar to that of extant representatives. Furthermore, the combination of a small number of known extant species and the often poor preservation of fossil representatives has greatly hindered the understanding of fossil psychopsids. The ambiguous diagnoses of many fossil psychopsids have resulted in potential confusion withother Mesozoic neuropterans (Andersen 2001; Makarkin and Archibald 2003). More evidence is needed to further the knowledge of fossil psychopsids. In recent years, many Mesozoic psychopsids described from Asia (particularly from Russia and China) have drawn increased attention to fossil psychopsids. In this paper, we describe a new genus and species of Psychopsidae from the Early Cretaceous Yixian Formation of Huangbanjigou Village, Liaoning Province, China.

**Table 1.** Fossil species currently ascribed to the family Psychopsidae.

	*Species*	*Age*	*Locality*
1	*Triassopsychops superbus* Tillyard, 1922	Late Triassic (Carnian)	Denmark Hill, Queensland, Australia
2	*Archepsychops triassicus* Tillyard 1919	Late Triassic (Carnian)	Denmark Hill, Queensland, Australia
3	*Apeirophlebia grandis* Handlirsch, 1906	Early Jurassic (Early Toarcian)	Dobbertin, Germany
4	*Cretapsychops decipiens* Penget al., 2010	Middle Jurassic (Aalenian/Bajocian)	Daohugou, Inner Mongolia, China
5	*Beipiaopsychops triangulatus* Hong, 1983	Middle Jurassic (Aalenian/Bajocian)	Haifanggou, Liaoning, China
6	*Sinopsychops chengdeensis* Hong, 1982	Middle Jurassic (Aalenian/Bajocian)	Chengde, Hebei, China
7	*Calopsychops extinctus* Panfilov, 1980	Late Jurassic (Oxfordian/Kimmeridgian)	Karatau, Kazakhstan
8	*Propsychops karatavicus* Panfilov, 1980	Late Jurassic (Oxfordian/Kimmeridgian)	Karatau, Kazakhstan
9	*Angaropsychops sinicus* Hong in Wang, 1980	?Early Cretaceous	Heishangou, Liaoning, China
10	*Kagapsychops aranea* Fujiyama, 1978	Early Cretaceous (Valanginian/Barremian)	Kuwajima, Japan
11	*Angaropsychops turgensis* Martynova, 1949	Early Cretaceous (Hauterivian)	Turga, Transbaikalia, Russia
12	*Psychopsites rolandi* Jepson et al., 2009	Early Cretaceous (Hauterivian)	Lower Weald Clay, Wealden, England
13	*Valdipsychops minimus* Jepson et al., 2009	Early Cretaceous (Hauterivian)	Lower Weald Clay, Wealden, England
14	*Baisopsychops lambkini* Makarkin, 1997	Early Cretaceous (pre-Barremian)	Baissa, Transbaikalia, Russia
15	*Epipsychopsis fusca* Makarkin, 2010	Early Cretaceous (pre-Barremian)	Baissa, Transbaikalia, Russia
16	*Epipsychopsis variegata* Makarkin, 2010	Early Cretaceous (pre-Barremian)	Baissa, Transbaikalia, Russia
17	*Undulopsychopsis alexi* gen. et sp. n.	Early Cretaceous (Barremian)	Huangbanjigou, Liaoning, China
18	*Cretapsychops corami* Jepson et al., 2009	Early Cretaceous (Barremian)	Upper Weald Clay, Wealden, England
19	*Micropsychops parallelus* Jepson et al., 2009	Early Cretaceous (Barremian)	Upper Weald Clay, Wealden, England
20	*Valdipsychops brigidae* Jepson et al., 2009	Early Cretaceous (Barremian)	Upper Weald Clay, Wealden, England
21	*Valdipsychops logunovi* Jepson et al., 2009	Early Cretaceous (Barremian)	Upper Weald Clay, Wealden, England
22	*Valdipsychops proudlovei* Jepson et al., 2009	Early Cretaceous (Barremian)	Upper Weald Clay, Wealden, England
23	*Valdipsychops maculosus* Jepson et al., 2009	Early Cretaceous (Barremian)	Upper Weald Clay, Wealden, England
24	*Pulchroptilonia espatifata* Martins-Neto, 1997	Early Cretaceous (Aptian)	Araripe Basin, Brazil
25	*Putzneura parcimoniosa* Martins-Neto & Rodrigues, 2010	Early Cretaceous (Aptian)	Araripe Basin, Brazil
26	*Litopsychopsis burmitica* Engel & Grimaldi, 2008	Early Cretaceous (Albian)	Burmese amber
27	*Embaneura vachrameevi* G. Zalessky, 1953	Late Cretaceous (Cenomanian)	Emba, Kazakhstan
28	*Grammapsychops lebedevi* Martynova, 1954	Late Cretaceous (Cenomanian)	Kem’ River, Siberia, Russia
29	*Kagapsychops continentalis* Makarkin, 1994	Late Cretaceous (Turonian)	Kzyl-Zhar, Kazakhstan
30	*Arctopsychops zherikhini* Makarkin, 1994	Late Cretaceous (Turonian)	Arkagala, NE Siberia, Russia
31	*Propsychopsis helmi* Krüger, 1923	Eocene (Lutetian/Bartonian)	Baltic amber
32	*Propsychopsis hageni* MacLeod, 1971	Eocene (Lutetian/Bartonian)	Baltic amber
33	*Propsychopsis lapicidae* MacLeod, 1971	Eocene (Lutetian/Bartonian)	Baltic amber
34	*Miopsychopsis relicta* Makarkin, 1991	Late Eocene/Early Oligocene	Amgu, Sikhote-Alin, Russia
35	*Miopsychopsis sikhotensis* Makarkin, 1991	Late Eocene/Early Oligocene	Amgu, Sikhote-Alin, Russia

## Material and methods

The specimen described herein is from the Yixian Formation of Huangbanjigou Village, Shangyuan County, Beipiao City, western Liaoning Province, northeastern China. The principal fossil-bearing layers in Huangbanjigou locality are silty mudstone, yellowish to grayish, rich in insects, fish and plants (Chen et al. 2005). The age of these fossil-bearing strata in Sihetun area (including Huangbanjigou) is considered to be well supported by radiometric dating as Early Cretaceous (Middle/Late Barremian), from 126.1 ± 1.7 to 124.6 ± 0.1 Ma (e.g., Swisher et al. 1999; Wang et al. 2001b; Chen et al. 2004; Yang et al. 2007), although the upper-most beds of Huangbanjigou locality have an Early Aptian age, 123.3 ± 0.5 – 122.8 ± 1.6 Ma (Wang et al. 2001a; Yang et al. 2007). The specimen is deposited in the Chaoyang Bird Fossil National Geopark, Chaoyang City, Liaoning Province, China.

The material was examined using a Leica MZ12.5 dissecting microscope. The photographs were taken using a Nikon D100 digital camera coupled to a Nikkor 105mm macro lens, and final photographs were adjusted by using Adobe Photoshop 4.0 image-editing software. All line drawings were drawn made directly using CorelDraw 12 graphic software.

We follow the traditional (*sensu* Wootton 2003) venational terminology of Comstock (1918) with the recent interpretation of Oswald (1993b) and Archibald and Makarkin (2006). The abbreviations used in the text are: C, costa; Sc, subcosta; hv, humeral veinlet; R, radius; R1, first branch of R; Rs, radial sector; Rs1, basal-most branch of Rs; M, media; MA, media anterior; MP, media posterior; Cu, cubitus; CuA, cubitus anterior; CuP, cubitus posterior; 1A–2A, first to second anal veins.

## Systematic palaeontology

### Order Neuroptera Linnaeus, 1758. Family Psychopsidae Handlirsch, 1906

#### Genus Undulopsychopsis gen. n.

urn:lsid:zoobank.org:act:B718D980-A2EB-4293-B2CD-EF6C58E8F53E

http://species-id.net/wiki/Undulopsychopsis

##### Type species.

*Undulopsychopsis alexi* sp. n.

##### Diagnosis.

Forewings: costal gradate series absent; branches of Rs dichotomously branched; Rs1 multi-branched, pectinate with branches directed anteriorly; M forked far distal to origin of Rs; CuP dichotomously branched. Hind and outer margins of both wings undulate.

##### Etymology.

The generic name is derived from the Late Latin*undula* (meaning a small wave, refers to its undulate wing margins) and *Psychopsis* (the type genus of the family). The gender is feminine.

##### Remarks.

This new genus differs from all other psychopsids by possessing undulate outer and hind margins of both wings. The combination of the following forewing character states is also characteristic: no costal gradate series; branches of Rs dichotomously branched; the basal-most branch of Rs multi-branched, and M forked far distal to the origin of Rs. The new genus has scarce costal crossveins, which are not arranged in gradate series, in contrast to the genera *Grammapsychops*, *Miopsychopsis* Makarkin, 1991, *Baisopsychops* Makarkin, 1997, *Cretapsychops* Jepson et al., 2009 and *Epipsychopsis* Makarkin, 2010. *Undulopsychopsis* gen. n.possesses the dichotomously branched branches of Rs; this condition is also present in the following psychopsid genera: *Triassopsychops*, *Angaropsychops* Martynova, 1949, *Psychopsites* Jepson et al., 2009, *Valdipsychops* Jepson et al., 2009, *Epipsychopsis*, *Pulchroptilonia*, *Putzneura* Martins-Neto & Rodrigues, 2010, *Kagapsychops*, *Grammapsychops*, and *Embaneura*. Among these the new genus is most similar to those genera which have the multi-branched Rs1 and M forked far distal to the origin of Rs. This combination is present only in the genus *Kagapsychops*. The type species of this genus (*Kagapsychops aranea* Fujiyama, 1978) is fragmentary and poorly preserved, but *Kagapsychops continentalis* Makarkin, 1994 is well-preserved (although incomplete). *Undulopsychopsis* gen. n.clearlydiffers from *Kagapsychops* by being a much smaller size (the forewing of the former is approximately twice shorter than that of the latter), and the absence of the gradate series of crossveins in the radial space. Other fossil psychopsids, for example *Propsychopsis* Krüger, 1923, *Litopsychopsis* Engel & Grimaldi, 2008 and *Micropsychops* Jepson et al., 2009 differ strongly from the new genus by having mostly unbranched veins of Rs before end-twigging and several long gradate series of crossveins in the radial space. 

#### Undulopsychopsis alexi sp. n.

urn:lsid:zoobank.org:act:29E097D3-A80A-48B2-88D7-8240349F17D0

http://species-id.net/wiki/Undulopsychopsis_alexi

Figs 1 [Fig F2] 3

##### Material.

Holotype CYNB044, a well-preserved specimen, with body partially preserved and four wings overlapping pairwise.

##### Diagnosis.

As for the genus.

##### Description.

Body: only partial thorax preserved. Pronotum sub-rectangular, 1.2 mm long, 2.8 mm wide, suffused with many long hairs. Mesonotum 3 mm long, 3.5 mm wide, with some long hairs laterally.

Forewing (Fig. 3) subtriangular, 21.5 mm long, 12.3 mm wide. Costal space broad throughout; subcostal veinlets forked; humeral veinlet slightly recurrent, branched. Subcostal space much broader than R1 space. R1 space narrow. Sc and R1 close distally but not fused. Rs with 10 primary branches, branches of Rs dichotomously branched; Rs1 pectinately branched with branches directed anteriorly. M appears originating from R, forked far from origins of Rs1. MA and MP probably simple (their terminal parts not preserved). Cu forked near wing base. CuA pectinately branched distal to fork of M. CuP multi-branched, dichotomous. Anal area well-developed. 1A long, dichotomously branched. 2A multi-branched (incompletely preserved). Only few crossveins detected: costal space basally with scarce crossveins, not forming gradate series; subcostal space with 4 crossveins preserved; R1 space with 5 crossveins preserved; medial space with 2 crossveins preserved. Veins covered with dense hairs, particularly long basally. Trichosors distinct. Wing membrane in general brownish; colour pattern consists mainly of two pale transverse zigzagged bands which are proximally darker than other portions of wing; indistinctly mottled basally and apically. Wing margin haired; hind and outer margins undulate.

Hind wings almost entirely hidden under forewing, about 16.5 mm long as preserved, 10 mm wide. Venation very poorly preserved; no details visible. Outer margin undulate.

**Figure 1.**
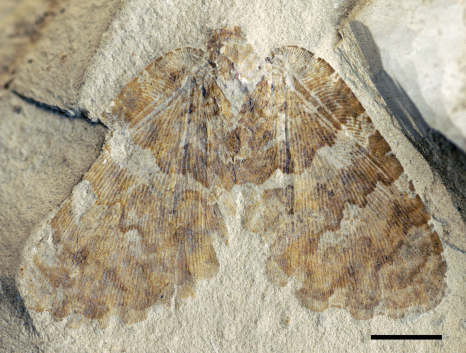
*Undulopsychopsis alexi* gen. et sp. n. The holotype CYNB044. Photograph. Scale bar = 5 mm.

**Figure 2. F2:**
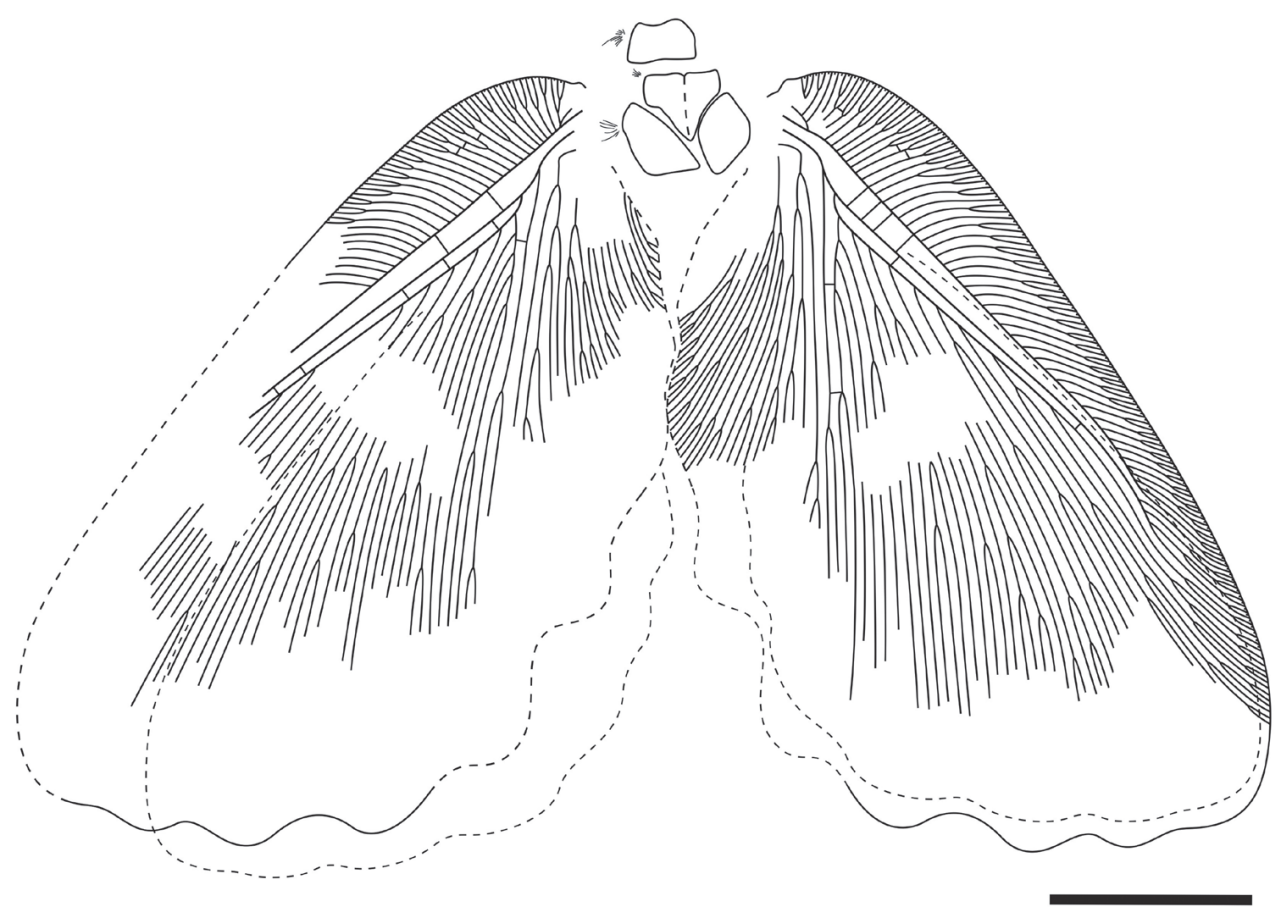
*Undulopsychopsis alexi* gen. et sp. n. The holotype CYNB044. Drawing. Scale bar = 5 mm.

**Figure 3.**
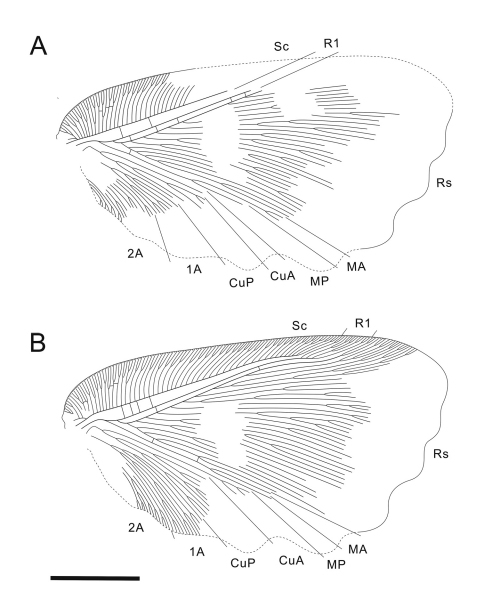
*Undulopsychopsis alexi* gen. et sp. n. The forewing venation of the holotype CYNB044. **A** left forewing (converted to the right) **B** right forewing. Scale bar = 5 mm.

##### Etymology.

The specific name is named in honor of the distinguished Russian paleoentomologist Prof. Alexandr (‘Alex’) Rasnitsyn.

##### Type locality and horizon.

Yixian Formation, Huangbanjigou Village, Shangyuan County, Beipiao City, Liaoning Province, China.

## Discussion

Based on the configuration of the venation in the radial space of the forewing, fossil psychopsids can be divided into two groups. One group includes the taxa with simple branches of Rs, the majority of which are not branched before end-twigging. This group is represented by the genera *Propsychopsis*, *Baisopsychops*, *Cretapsychops*, *Micropsychops* and *Litopsychopsis*. The crossveins in these genera are usually arranged in one to two gradate series in the costal space, and two to four long gradate series in the radial space. They occur in the Cretaceous and Eocene; all extant genera belong to this group as well.

The other group includes the taxa which have the branches of Rs dichotomously branched, and often the basal-most branch of Rs multi-branched. Representative genera of this group are the earliest psychopsid *Triassopsychops*, and other Mesozoic psychopsids, e.g., *Angaropsychops*, *Grammapsychops* and *Kagapsychops* (see complete list above). They possess numerous radial crossveins, arranged in many short gradate series (often irregular), and usually no costal gradate series. *Undulopsychopsis* gen. n. belongs to the latter group. It is preliminarily assigned to Psychopsidae, as its Sc and R1 are not fused apically, and the costal space is broad, although it almost lacks crossveins. The latter feature, and the multi-branched Rs1 and 1A are shared by this genus with another Mesozoic psychopsoid family Osmylopsychopidae (especially with its type genus) known from the Triassic of Australia and Central Asia (Lambkin 1992; Shcherbakov 2008). However, in the family Osmylopsychopidae Sc and R1 are clearly fused apically. Some genera currently ascribed to Psychopsidae also have venation similar to that of Osmylopsychopidae (e.g., Sinopsychops Hong, 1982; *Grammapsychops*). Unfortunately, the majority of these are either fragmentary or incompletely described and are in need of re-examination. Therefore, until the revision of psychopsoids has been completed, we consider all species enumerated in Table 1 as tentatively belonging to Psychopsidae.

Previously, only four species have been recorded from the Mesozoic of China, i.e., *Angaropsychops sinicus* Hong in Wang, 1980 (probably from the Early Cretaceous Yixian Formation), *Sinopsychops chengdeensis* Hong, 1982, *Beipiaopsychops triangulatus* Hong, 1983, and *Cretapsychops decipiens* Peng et al., 2010 (all from the Middle Jurassic Jiulongshan Formation). *Undulopsychopsis* gen. n. is the fifth representative of the Chinese psychopsids found from the different locality (Huangbanjigou). It is characterized by the undulate wing margin, a unique character state among known Psychopsidae, and the forewing venation that is not typical for this family compared with most other genera of Psychopsidae.

## Supplementary Material

XML Treatment for Undulopsychopsis

XML Treatment for Undulopsychopsis alexi
